# How to Prevent, Reduce, and Treat Severe Post Sympathetic Chain Compensatory Hyperhidrosis: 2021 State of the Art

**DOI:** 10.3389/fsurg.2021.814916

**Published:** 2022-01-03

**Authors:** Federico Raveglia, Riccardo Orlandi, Angelo Guttadauro, Ugo Cioffi, Giuseppe Cardillo, Gerardo Cioffi, Marco Scarci

**Affiliations:** ^1^Department of Thoracic Surgery, San Gerardo Hospital, Monza, Italy; ^2^Department of Medicine and Surgery, Istituti Clinici Zucchi Monza, University of Milano-Bicocca, Milan, Italy; ^3^Department of Surgery, University of Milan, Milan, Italy; ^4^Department of Thoracic Surgery, Azienda Ospedaliera San Camillo-Forlanini, Roman, Italy; ^5^Department of Sciences and Technologies, University of Sannio, Benevento, Italy

**Keywords:** primary hyperhidrosis, compensatory hyperhidrosis, thoracic sympathicotomy, thoracic sympathectomy, prevention

## Abstract

The role of thoracic surgery in the management of hyperhidrosis is well-known and thoracoscopic sympathetic interruption is commonly accepted as being the most effective treatment. However, some concerns still remain regarding the potential to develop compensatory hyperidrosis (CH), the most troublesome and frequent side effect after surgery and its management. Compensatory hyperidrosis prevention may be achieved by identifying subjects at higher risk and/or targeting nerve interruption level on the base of single patient characteristics gathered during the preoperative survey. Furthermore, the surgical treatment may consist of different techniques aimed at reversing the effects of previous sympathetic interruption. To predict CH after sympathectomy, the most interesting proposals in recent literature are a temporary thoracoscopic sympathetic block and the introduction of new and targeted preoperative surveys. If the role of nerve clipping technique vs. the definitive cutting is still intensely under debated, new approaches have been recently proposed to reduce the incidence of CH. In particular, extended sympathicotomy has been described as an alternative to overcome severe forms. Last, among the techniques developed to reverse sympathetic interruption effect, diffuse sympathicotomy (DS) and microsurgical sympathetic trunk reconstruction represent advances in this field. An all-round review of these topics is strongly needed. Our aim is to cover all the above issues point by point. Although sympathectomy represents a small part of thoracic surgery, we believe that it is worthy of interest because of the profound effect that complications for a benign condition can have on patients.

## Introduction

Primary hyperhidrosis (PH) (Table 1) is a condition of abnormal sweating compared with physiological body thermoregulation consisting of excessive and uncontrollable activation in the eccrine sweat glands of definite skin areas, such as the craniofacial region, axilla, hands, and feet (1, 2). Although it is not a life-threatening disease, hyperhidrosis interest 1–3% of population worldwide determining severe trouble in the social, mental, and working life of an individual, and leading to a decreased quality of life (QoL). PH etiology is still unclear but is commonly accepted that the abnormal sweating is associated with sympathetic nervous dysfunction. The treatment modality for PH includes a range of topical or systemic medical agents, psychotherapy, and surgery (1, 3, 4). The role of thoracic surgery in its management is well known and thoracoscopic sympathetic interruption is commonly accepted as the most effective and definitive treatment when conservative ones have failed (5–8). However, some deep concerns are still present and regarding, in particular, the onset and management of the most feared, troublesome, and frequent side effect that may arise after surgery: compensatory hyperidrosis (CH).

**Table 1:** Summary table of abbreviations.

**Full text**	**Abbreviation**
Compensatory hyperhidrosis	CH
Primary hyperhidrosis	PH
Society of thoracic surgeons	STS
Hyperhidrosis disease severity scale	HDSS
Hyperhidrosis impact questionnaiere	HHIQ
Dermatology quality of life index	DLQI
Numeric analogic scale	NAS
Diffuse sympathicotomy	DS
Sympathetic nerve reconstruction	SNR
International society of sympathetic surgery	ISSS
International hyperhidrosis society	IHS

Despite advances in surgical techniques, CH remains the most troublesome postoperative side effect, which occasionally causes patients to regret choosing surgery and surgeons to hesitate to suggest sympathectomy as upfront treatment for PH, especially for craniofacial hyperhidrosis (1, 9–11).

Postoperative CH is reported in 50–90% of patients depending on the Authors and is experienced “severe” in as many as 35% patients (2, 5, 12). CH pathogenesis is poorly understood but has been hypothesized that a dysfunctional reflex arc from the sympathetic nervous system to hypothalamus could be the main factor causing uncontrollable, unexpected, and massive sweat in other parts of the body (13, 14). Important predisposing factors are the block of the sympathetic nerve at the T2 level or, possibly, at multiple levels (15).

Currently, there is no effective treatment for CH after thoracic sympathectomy. Indeed, there have been several attempts to reduce CH, such as both preoperative and postoperative strategies. Among the preoperative one, limited level sympathectomy (16) or lower level sympathectomy (17) have been the most adopted.

Compensatory hyperhidrosis onset may be prevented or mitigated through several actions ranging from prevention to re-surgery. Prevention may be obtained by identifying subjects at higher risk and/or targeting nerve interruption level on the basis of single patient characteristics. On the contrary, surgical treatment may consist of techniques aimed to reverse the effects of previous sympathetic interruption.

In the past decade, no one of these solutions took over, and CH is still the major cause of dissatisfaction for patients. However, in the past few years, some innovative approaches have been described and proposed. In this article, we are going to summarize insights in preoperative and postoperative management of patients who have undergone sympathetic interruption or presenting CH, with the aim to give a snapshot of the state of the art and promote valuable innovations.

## Methods

Currently, there are two well established strategies to avoid or deal with CH: (1) optimization targeting of sympathetic chain level and (2) chain clipping and subsequent clips removal.

In 2017, Sang and co-workers published an outstanding review to identify the appropriate level of nerve chain interruption in terms of both efficacy in PH management and CH onset reduction for palmar hyperhidrosis (15). Their background was the evidence that different levels of nerve ablation correspond to different outcomes. Authors results have led to the conclusions that T2 ablation should always be avoided and that T3 or T4 are the optimal targets. This article and many others do nothing except supporting the 2011 recommendations from the Society of Thoracic Surgeons (STS) that prompted the location of interruption of sympathetic chain based on patient's distribution of sweating (18). Ten years ago, the STS concluded that “although the level of interruption, based on the patient's distribution of the hyperhidrosis, remains controversial, expert consensus based on a standardized anatomic classification and careful literature review provides the framework for our suggested treatment strategies.” Today this criterion is still used despite the doubts expressed by the Authors themselves.

In 2002, Reisfeld and co-workers (19) recommended nerve clipping rather than permanent disruption based on acceptable results after clips removal in reducing CH. The unclipping technique is supported by alleged regeneration of the nerve as cause for CH reversing. This process is called nerve regeneration and has been advocated by many Authors supporting clipping technique.

However, without considering that de-clipping requires a further and more complex operation, Loscertales and co-workers (20) in a prospective experimental study clearly demonstrated the total absence of myelin and amyelin fibers in 10 days after unclipping, thus, refuting any nerve regeneration theory. Furthermore, Kara et al. in their series reported an unsuccessful clinical experience with patients affected by CH that were subsequently unclipped, concluding that (1) the basis for unclipping is empirical, (2) the International Society of Sympathetic Surgery (ISSS) states that unclipping acts as a placebo, and (3) unclipping does not help in CH management especially if not performed within a few days after the first surgery (21).

Therefore, since the techniques described above present many drawbacks and do not guarantee success, further strategies are needed. We have selected the most interesting articles describing innovative techniques and their results to prevent or control CH in patients affected by PH.

## Preoperative Measures Section

### Patient Tailored Preoperative Surveys

Nowadays, many questionnaires and scales for patients affected by PH evaluation are available. The most diffuse one is the Hyperhidrosis Disease Severity Scale (HDSS) from the International Hyperhidrosis Society (IHS). It is a disease-specific, quick, and easily understood 4-point scale that qualitatively investigate the severity of sweating in daily activities. The HDSS determines PH severity based on the extent of sweating-related discomfort. Its effectiveness has been verified in three remarkable studies describing a strong to moderate correlations with the Hyperhidrosis Impact Questionnaire (HHIQ), Dermatology Quality of Life Index (DLQI), and gravimetric sweat production measurements.

The Hyperhidrosis Impact Questionnaire, DLQI, and Hyperhidrosis Quality of Life Index (HidroQoL©) are other surveys mostly adopted by the dermatologist to measure QoL in patients affected by PH. They are mainly used to make diagnosis and investigate outcomes and satisfaction of the treatment. However, current questionnaires are not helpful in selecting patients who are more likely to develop CH after sympathectomy.

We have recently published an article (22) focused on novel techniques in surgical management of PH; in this study, we described the role of careful preoperative PH mapping by the use of an innovative numeric questionnaire. Our background was that (1) lowering the nerve interruption level decrease CH onset rate, (2) whole body PH severity grade is predictive for CH, and (3) CH onset is more likely and troublesome in that skin area already characterized by higher propensity to sweat.

Therefore, we introduced a preoperative NAS survey quantifying discomfort in each body area. CH risk was derived by adding 2 points to initial score of each area and then, calculating a whole-body median score. Then, the selective clipping was tailored to reduce CH risk as follows: in case of palmar or palmar-axillary PH, if median score was <7, nerve trunk was clipped at the superior margin of third and fourth rib. On the contrary, if estimation was >7, nerve trunk was clipped only at the upper margin of fourth rib. Cephalic PH was treated by one clip application at the lower margin of second rib if CH estimate was <7. Last, diffuse PH was treated by clips application at the upper margin of third, fourth, and fifth rib regardless of any CH estimate. We investigate our survey comparing the outcomes in terms of PH management, CH onset, and satisfaction of a patient with a group of patients treated by targeted nerve interruption for each location of PH, according to the ongoing STS recommendation.

Results showed that our interruption-level pattern tailored to preoperatively determined CH risk guarantees better outcomes. Further studies are needed to validate the questionnaire; however, our experience confirms that a patient tailored approach based on individual preoperative sweating pattern could be a successful method to prevent CH onset.

### Temporary Thoracoscopic Sympathetic Block Technique

Despite ongoing recommendations have already identified the optimal target level for nerve interruption for each skin area affected by excessive sweating, two main concerns still persist: (1) recommendations do not punctually indicate where interruption should be performed but usually suggest a range encompassing almost two intercostal spaces and (2) CH onset rate is still significant. In our opinion, the most likelihood cause is individual variability, therefore any techniques aimed to anticipate the occurrence and grade of CH in each single patient, should be encouraged.

With this goal in mind, Lee et al. in the past 10 years developed a procedure to obtain a temporary nerve block to simulate definitive sympathectomy and predict after surgery outcomes of patient (23). In particular, they introduced a thoracoscopic sympathetic block under local anesthesia. Under thoracoscopic guidance (2 mm camera, single port, and CO_2_ insulation), they blocked predetermined ganglia by the use of a percutaneous spinal needle injecting 10 ml of a mixture of ropivacaine, steroids, and epinephrine. A week after temporary blocks, patients were asked about their satisfaction in terms of PH management and CH onset. In case of good results, patients decided whether to undergo definitive sympathectomy or not. Their results were encouraging since the predictive procedure was safe and effective in the most of cases in terms of specificity (94.4%) and positive predictive rate (95.2%). This allowed the Authors to conclude that their method successfully offers patients the opportunity to experience effects of definitive surgery improving postoperative overall satisfaction.

Despite some bias, already highlighted by the Authors themselves, the procedure could be the corner stone in patients at higher risk for CH such as: cranio-facial cases, high body mass index (BMI), or diffuse hyperidrosis.

Since preoperative patient evaluation for CH risk determination is a constant evolving issue, as we will address in the next paragraphs, temporary predictive chain block could be proposed in selected patients to offer an experience on which they decide and therefore improving their postoperative satisfaction.

## Intraoperative Measures Section

### Extended Sympathectomy

As reported above, the most significant surgical innovations aimed to reduce CH have been represented by nerve interruption targeting and by clipping introduction. However, results are still not completely satisfactory.

In 2020, Han and co-workers presented their new sympathectomy for the prevention of severe CH (24). Their background was that (1) there is not association between the extent of sympathectomy and the onset of CH and (2) CH should occur because of the remaining nerve chain from the hypothalamic reflex arc. They based these statements from previous experiences by different Authors (10). They were also supported by some studies and experiences showing that very low nerve damages were not associated to any complications. Thus, they introduced the wide sympathectomy; in addition to conventional target level, they also performed sympathectomy from R5 to R12 by chain ablation. This procedure is called (full or partial) expanded sympathectomy. Their data were encouraging since comparing patients undergone partial-expanded and non-expanded sympathectomy with patients undergone full-expanded sympathectomy results showed a significancy in favor of the full-expanded technique as concerning both PH management and CH onset. They never recorded adverse effects correlated with this procedure. To conclude, this innovative surgical approach, reversing the current trend to reduce the number of nerve interruptions, could represent a successful alternative in lowering CH onset rate.

## Postoperative Measures Section

### Diffuse Sympathectomy

All the procedures and techniques previously described in this paper were focused on identifying patients at higher risk for CH; they are preventive solutions useful for lowering the postoperative excessive sweating onset by preoperative selection of patient. Instead, the opposite solution involves management of patients who have already developed CH following sympathectomy. Indeed, as already showed, ongoing methods to prevent CH are unsatisfactory and the innovative ones are still under investigation. Thus, it is mandatory to develop effective therapies to be proposed for a cure, or at least a QoL improvement of these patients. As of now, medical and non-invasive treatments for CH are unsuccessful in the most of severe cases, therefore, surgical innovations are desirable.

Moon and co-workers have recently published an article addressing this topic and entitled surgical treatment of compensatory hyperhidrosis (16). Their background is the common experience that sites affected by CH are generally thermoregulatory, non-glabrous skin regions of the trunk/back, buttocks, groin, and thighs that are not affected by PH before surgery. In particular, it is suggested that CH arises as a normal thermoregulatory effector and becomes upregulated in a context of normal heat dissipation. Therefore, they managed to block these reflex sweating responses by interrupting the segmental origins of the sympathetic nervous system. They were encouraged by some series reported in literature showing that expanded sympathectomy was rarely affected by any complication.

Since the sympathetic dermatomes overlap each other and are hardly identifiable, they initially proposed a limited diffuse sympathicotomy (DS) for denervation in CH. Limited DS comprised the nerve interruption at right R5, R7, R9, and R11 and left R5, R6, R8, and R10 levels. Based on good outcomes and no complications, they replaced the limited DS with extended DS consisting of all level interruption (R5, R6, R7, R8, R9, R10, and R11 on both sides). Results are interesting and deserve discussion. First, it is remarkable that Authors could obtain an immediate and diffuse control of CH in 81% of case treated by DS. However, unfortunately, outcomes progressively got worse with 54% of cases presenting a recurrence at long-term follow-up.

Instead, results were much more successful in patients who experienced extended DS that at multivariate logistic regression analysis resulted an independent predictive factor for CH resolution. As well as zone of compensatory hyperhidrosis involvement (upper, middle, and lower zone together) were differently associated with persistent resolution.

Some biases and the small population enrolled make this study too weak for definitive conclusions; however, in our opinion this study sheds new lights on surgical management of CH.

### Microsurgical Sympathetic Nerve Reactivation

As reported in literature, postoperative CH management with a topic or systemic therapy is unlikely successful in terms of efficacy, duration, and overall satisfaction of a patient. Therefore, many attempts to develop a surgical technique suitable to stop or at least reduce CH have been proposed. However, at present, this kind of surgery is still at the embryonic level due to the controversial results and technical difficulty. Therefore, there are two possibilities to increase the use of surgery in this field currently: the introduction of new reconstructive techniques and the use of the latest generation technology.

As concerning new technologies, the most interesting issue is the advent of robotic microsurgery. Chang and co-workers (25) last year have reported their experience with robotic surgery for sympathetic nerve reconstruction (SNR). Their study is the second one in literature but is also the largest series available.

They adopted the traditional technique consisting of SNR using a sural nerve grafting; the innovation is the introduction of the robotic microsurgical approach instead of classic thoracotomy. The background has potentially better feasibility of a robot-assisted approach, thanks to the 3D vision and wrist-like movements. Authors reported the results in terms of surgical feasibility showing complete success in 7 cases. Despite short follow-up, they were able to observe interesting improvement in symptoms compared with pre-operative condition. Thus, they concluded that robotic approach provides better visualization and better movements in suturing with 8-0 nylons. However, further studies focused on clinical outcomes are mandatory.

As concerning new reconstructive techniques, Gebitekin and co-workers (26) in 2020 described an innovative approach instead of traditional nerve grafting that has not reached definitive positive results. Their background was that (1) Kuntz fibers are probably the most important factor for surgery failure in PH, (2) CH probably originated from a deficiency in negative feedback by efferent nerves, (3) intercostal nerves have fibers arriving from and going to the sympathetic chain, and (4) the negative feedback to hypothalamus could be restored activating an alternative pathway.

Thus, they developed the Gebitekin Technique consisting of constructing a parallel pathway to sympathetic chain with 2 healthy intercostal nerves accurately selected, prepared, and joined by a tension free anastomosis. Collecting data from their series of 15 cases, they obtained good results in terms of feasibility and clinical outcomes comprising symptoms improvements and QoL.

## Conclusions

This review originates form the growing interest on this topic probably due to the number of patients who resort to surgery for hyperhidrosis and patients already affected by compensatory sweating. This condition and the absence of evidence concerning both prevention and management of CH have prompted surgeons to develop new and alternative methods to improve sympathetic block outcomes and manage any compensatory sweating.

As concerning the newest insights, the past 2 years literature offers very interesting innovations (Table 2). In particular, (1) preoperative patient selection could be improved by the adoption of tailored surveys or temporary nerve blocks simulating definitive surgery, (2) nerve interruption could be improved by the adoption of an extended technique involving the lowest thoracic ganglia, and (3) CH could be surgically managed by the introduction of diffuse sympathectomy or robotic nerve reconstruction or Gebitekin technique.

**Table 2:** Mini-review results overview.

**References**	**Title**	**Journal**	**Technique**	**Results**
Raveglia et al. (22)	Anatomical clipping of sympathetic nerve to reduce compensatory sweating in primary hyperhidrosis: a novel technique.	Shanghai Chest. (2019) 3:28	Patient tailored preoperative survey	Patient retrospectively enrolled = 460 320 palmar, 100 cephalic and 40 diffuse HP. Complications 7% (3 Bernard -Horner syndromes, 1 pleuritis, 24 PNX requiring chest tube, 1 hematoma, 2 chronic pain cases and 2 wing scapula). Severe CH (VAS > 7) = 6.2% of cases
Lee et al. (23)	Thoracoscopic sympathetic block to predict compensatory hyperhidrosis in primary hyperhidrosis.	J Thorac Dis. (2021) 13:3509–17.	Temporary sympathetic block	Patient retrospectively enrolled = 107 CH rate = 29.9% Predictive procedure specificity = 94.4% Predictive procedure sensitivity = 33.3%
Han et al. (24)	New sympathicotomy for prevention of severe compensatory hyperhidrosis in patients with primary hyperhidrosis.	J Thorac Dis. (2020) 12:765–72.	Extended sympathectomy	Patient retrospectively enrolled = 21 Expanded sympathicotomy = 67 cases (28 partial, 39 full)- Conventional = 145 cases Craniofacial hyperhidrosis: no difference concerning CH between expanded and conventional technique (*p* = 0.474) Palmar hyperhidrosis: significative difference concerning CH between expanded and conventional technique (*p* = 0.001) CH degree more severe in partial than full technique (craniofacial, *p* = 0.002; palmar, *p* < 0.001)
Moon et al. (16)	Surgical treatment of compensatory hyperhidrosis: Retrospective observational study.	Medicine. (2020) 99:e22466.	Diffuse sympathectomy	Patient retrospectively enrolled = 44 CH immediate resolution = 81% cases At 22.7 months follow-up CH resolved in 46% of cases Persistent CH resolution was independently predicted by extended DS (OR 25.67, 95% CI, 1.78–1047.6; *p* = 0.036 No side effects.
Chang et al. (25)	Microsurgical robotic suturing of sural nerve graft for sympathetic nerve reconstruction: a technical feasibility study.	J Thorac Dis. (2020) 12:97–104.	Robotic microsurgical nerve reactivation	Patients enrolled = 7 Median operating time = 10.5 h Median length of stay = 4 days No mortality. Minor complication (PNX)
Gebitekin et al. (26)	Intercostal nerve reconstruction for severe compensatory hyperhidrosis: the Gebitekin techinique	Ann Thorac Surg. (2021) 111:e443–6. https://doi.org/10.1016/j.athoracsur.2020.11.067	Intercostal nerve reconstruction	Patients enrolled = 15 Procedures performed = 30 Median surgery time = 90 min Excellent CH improvement = 46.7% Satisfactory CH improvement = 46.7 Unchanged = 6.6% No PH recurrence

Moreover, these procedures could be integrated each other; for instance, the temporary nerve block could be offered to patients who are more likely to develop CH on the basis of a tailored survey.

Unfortunately, all the technique described in this review are encouraging but not definitive and need further studies. Our aim is the diffusion of knowledge of new technologies between the thoracic surgeon community so that other centers, based on what already experienced, could start new trails and add their data to consolidate what at the moment are only intuitions.

## Author Contributions

All authors listed have made a substantial, direct, and intellectual contribution to the work and approved it for publication.

## Conflict of Interest

The authors declare that the research was conducted in the absence of any commercial or financial relationships that could be construed as a potential conflict of interest.

## Publisher's Note

All claims expressed in this article are solely those of the authors and do not necessarily represent those of their affiliated organizations, or those of the publisher, the editors and the reviewers. Any product that may be evaluated in this article, or claim that may be made by its manufacturer, is not guaranteed or endorsed by the publisher.
